# Disrupting Immune Regulation Incurs Transient Costs in Male Reproductive Function

**DOI:** 10.1371/journal.pone.0084606

**Published:** 2014-01-06

**Authors:** Virginia Belloni, Gabriele Sorci, Eugenio Paccagnini, Romain Guerreiro, Jérôme Bellenger, Bruno Faivre

**Affiliations:** 1 Biogéosciences, CNRS UMR 6282, Université de Bourgogne, Dijon, France; 2 Department Tropical Medicine, University of Tulane, New Orleans, Louisiana, United States of America; 3 Dipartimento di Biologia Evolutiva, Universita’ di Siena, Siena, Italia; 4 Lipides Nutrition Cancer, INSERM UMR 866, Université de Bourgogne, Dijon, France; Institut Pasteur de Lille, France

## Abstract

**Background:**

Immune protection against pathogenic organisms has been shown to incur costs. Previous studies investigating the cost of immunity have mostly focused on the metabolic requirements of immune maintenance and activation. In addition to these metabolic costs, the immune system can induce damage to the host if the immune response is mis-targeted or over-expressed. Given its non-specific nature, an over-expressed inflammatory response is often associated with substantial damage for the host. Here, we investigated the cost of an over-expressed inflammatory response in the reproductive function of male mice.

**Methodology/Principal Findings:**

We experimentally blocked the receptors of an anti-inflammatory cytokine (IL-10) in male mice exposed to a mild inflammatory challenge, with each treatment having an appropriate control group. The experiment was conducted on two age classes, young (3 month old) and old (15 month old) mice, to assess any age-related difference in the cost of a disrupted immune regulation. We found that the concomitant exposure to an inflammatory insult and the blockade of IL-10 induced a reduction in testis mass, compared to the three other groups. The frequency of abnormal sperm morphology was also higher in the group of mice exposed to the inflammatory challenge but did not depend on the blockade of the IL-10.

**Conclusions:**

Our results provide evidence that immune regulation confers protection against the risk of inflammation-induced infertility during infection. They also suggest that disruption of the effectors involved in the regulation of the inflammatory response can have serious fitness consequences even under mild inflammatory insult and benign environmental conditions.

## Introduction

Ecological immunology has emerged as a discipline in the late 90^th^ with the fundament that anti-parasite defenses cannot confer maximal protection because of the costs associated with immunity [1], [2]. Early studies focused on i) the metabolic costs of immune maintenance and activation [3]–[6]; ii) the life history consequences of immune activation [7]–[9]. Even though not all studies converged towards measurable costs of immunity [10], [11], an overall picture emerged consistent with the view that the expression of immune defenses is constrained by the associated costs [12], [2]. This early work mostly neglected the costs that are induced by a misdirected or an overreacting immune response (but see [13], [14]). Both antigen-specific and antigen non-specific immune responses can be erroneously directed against the host cells and tissue potentially generating devastating damage [15]. Immune disorders are among the most prevalent human diseases and again involve both antigen-specific and antigen non-specific mechanisms. Autoimmunity and immunopathology have understandably attracted considerable attention from biomedical scientists, but we still largely ignore how they can shape the evolution of immune defenses and parasite exploitation strategies [16]–[20]. This gap on our knowledge on the fitness consequences of immunopathology has started to be filled with studied conducted on laboratory and free-ranging animals [21]–[24].

Inflammation is one of the most important components of immune protection. Upon entering in contact with pathogen antigens, cells of the inflammatory response release signaling molecules (pro-inflammatory cytokines) which amplify the response by recruiting other macrophages and granulocytes to the infection site. These cytokines can therefore be seen as a turn on signal. As long as effector cells do produce these signaling molecules the inflammatory process continues. To prevent inflammatory damage, another set of signaling molecules have the function of turning the signal off. These regulatory, anti-inflammatory cytokines play therefore an essential role to maintain organism homeostasis during the infection, and studies on humans and mice have shown the involvement of poor immune regulation in several inflammatory diseases [15].

Aging is accompanied by profound changes in the functioning of the immune system [25]–[27]. The number of naïve T lymphocytes declines with age making elderly less able to respond to novel antigens [25], [28]. Senescent immune profiles are usually also characterized by an increase of inflammatory markers [29], [30], whose cause might be an impaired regulation [31].

Recently, a few papers have addressed the fitness consequences of impaired immune regulation of the inflammatory response using a phenotypic manipulation that blocks the receptors of one of the principal anti-inflammatory cytokines (IL-10). These studies have shown that disrupting the anti-inflammatory response exacerbates the cost of infection in mice infected with *Plasmodium chabaudi* parasites [21]. Similarly, using bacterial lipopolysaccharide as a proxy of bacterial infection, Belloni et al. [22] showed that mice temporarily impaired in their anti-inflammatory response suffered substantial survival costs, but only when in advanced age.

To our knowledge, reproductive costs of failure of the anti-inflammatory network have never been investigated yet, in spite of germ line being particularly sensitive to infection and free radical species released during the oxidative burst [32]. Infection and inflammation can reduce male fertility, and inhibition of testicular steroidogenesis and disruption of spermatogenesis have been reported in animals challenged by bacterial lipopolysaccharides (LPS) [33]. Several cytokines have direct effects on testicular cell functions, and a number of these cytokines are produced within the testis even in the absence of inflammation or immune activation. Consequently, local or systemic up-regulation of cytokine expression during infection may contribute to the disruption or testicular function and fertility [34], [35].

The aim of this study was to assess the fitness consequences of an impaired regulation of the inflammatory response, focusing on the male reproductive function. We disrupted the regulation of the inflammatory response by injecting male mice with a monoclonal antibody that blocks the IL-10 receptors. Mice were then injected with *Escherichia coli* lipopolysaccharides as to elicit an inflammatory response. Both treatments had an appropriate control and the entire experiment was replicated using two age classes (3 and 17 month old).

We predicted that i) mice with an impaired IL-10 response should pay an exacerbated cost when exposed to the inflammatory insult; ii) old mice with a dis-regulated anti-inflammatory response should pay a higher cost than young individuals.

## Materials and Methods

### Ethical Note

The experiment has been conducted in compliance and has received the agreement of the Animal Care and Ethical Committee of the Université de Bourgogne, Dijon (protocol # 6904). During the course of the experiment, six out of 77 individuals died (7.8%). Mice were monitored daily to assess signs of extreme distress. Two criteria of endpoints were adopted for this study because they well describe the sickness behavior usually induced by a LPS injection: rapid weight loss (>20% of initial body mass) and prolonged impaired ambulation. None of the animals was in such conditions. For instance, the mean weight loss of the six animals that died was 5.42% (min = 0.54, max = 8.53).

At the end of the experimental period (day 9), five animals per group and per age (total = 40 mice) were euthanized with an overdose injection of pentobarbital. Similarly, four months after the end of the experiment, other 20 mice belonging to the young age group were euthanized with an overdose injection of pentobarbital.

### Animals and Reagents

Young (3 month old) and old (17 month old) male C57BL/6 mice were purchased from Janvier (Laval, France). Upon arrival at the laboratory, animals were individually housed in Plexiglas cages and kept in an air-conditioned room (temperature 21±1°C, relative humidity 60±10%) with a 12-hours light/dark cycle. Pellet food and tap water were provided *ad libitum*.

### Experimental Design

After two-week acclimation in the lab, young (n = 40) and old mice (n = 37) were randomly assigned to one of four groups. At day 1, the group “anti-IL-10R” received an intraperitoneal injection of 20 µg of monoclonal anti-IL-10 receptor antibodies (1B1.3a; BD PharMingen), whereas the group “IgG” received an intraperitoneal injection of 20 µg of rat IgG1 antibodies (Sigma) for control. At day 2, half of the males in each group were intraperitoneally injected with a solution of lipopolysaccharides (LPS) from *Escherichia coli* (serotype 055:B5, Sigma) at a dose of 0.05 mg/kg, whereas the other half were injected with a phosphate buffered solution (PBS) for control. This LPS dose is more than 500-fold and 30-fold lower than the 50% lethal dose of LPS in 2-month and 24 month old C57BL/6 mice respectively (Tateda et al. 1996). No injection was applied at day 3. This scheme was repeated three times, each three days, and the entire experiment covered a 9 day period. Body mass (±0.01 g) was measured at day 1, 3 and 9. Mortality rate was recorded every day for the duration of the experiment. To evaluate long term effects, differences in body mass and testis mass were measured in young individuals (n = 20) four months after the end of the experiment. Because of the mortality that occurred in the anti-IL-10R/LPS among old individuals, it was not possible to assess the long term effects of the treatments in old mice.

### Testis Mass

At the end of the experimental period (day 9), five mice per group were weighted (±0.01 g) and euthanized with a pentobarbital injection. Both testes and cauda epididymes were surgically removed.

Right and left testes were weighed separately immediately after removal with an electronic balance (±0.0001). The left testes and the left cauda epididymes were then placed in separate Petri dishes containing 2 ml TNE buffer (0.15 M NaCl, 0.01 M Tris–HCl, 0.001 M EDTA, pH 7.4) mixed with glycerol to a final concentration of 10% (v/v) and then minced with curved scissor, which allowed to assess sperm morphology separately for testes and epididymis. The larger fragments were allowed to settle and the sperm suspension was gently aspirated with a Pasteur pipette, transferred into 2 ml cryogenic vials, and frozen at −80°C until analysis.

### Sperm Morphology

To examine sperm morphology, a drop of the suspended sperm was placed on a glass slide, with a 22×32 mm coverslip, and observed under a phase contrast microscopy connected to a digital camera. Slides were inspected at 40× magnification and digital phase-contrast microscope images of intact sperm were collected. For each male, 100 spermatozoa were counted and screened.

Three parts of the sperm, namely the head, tail, and CD (cytoplasmic droplet) were examined. In particular, abnormal sperm head, including triangular, collapsed and hammer head and the presence of the hairpin at the neck were recorded. Sperm tail morphology was classified into four categories: straight tail, proximal bent tail (*i.e.*, the tail curved in the mid-piece), distal bent tail (*i.e.*, the tail angled at the end of the mid-piece), and coiled tail [36]. This information was synthetized using the proportion of sperm with one abnormal morphological trait and the proportion of sperm with multi-abnormalities. We also computed the teratozoospermia index (TZI; [37], [38]) as a the sum of the overall abnormal sperm (number of defects in the head, mid-piece and tail) divided by the number of morphologically abnormal spermatozoa.

### Statistical Analyses

Differences in survival rate were analyzed with a Log-rank test. A linear mixed model was used to assess the effect of the treatments and age on changes in body mass, with individual identity declared as a random variable and a normal distribution of errors. Testis mass (log-transformed), the percentage of sperm with abnormal morphologies and multi-abnormalities (square-root transformed) and the TZI were analyzed using ANOVAs and MANOVAs. The model exploring the variation in testis mass also included body mass measured to day 9 to take into account for any allometric relationship between testis and body mass. However, since body mass was never significant it was removed from the final model.

## Results

### Survival

**Figure 1:**
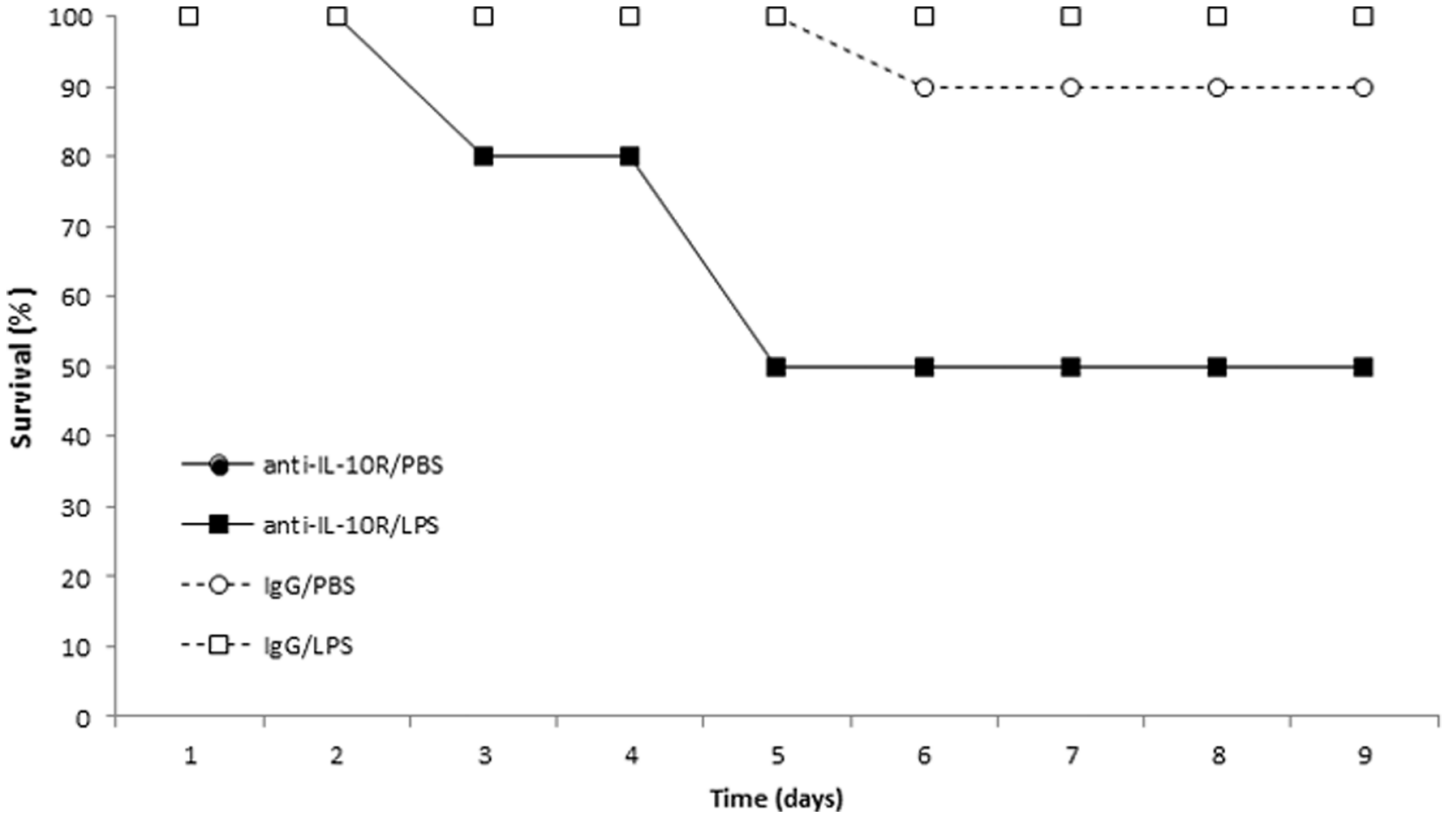
Survival of old male mice (17 month old) according to the four experimental treatments.

During the course of the experiment no young mice died (0/40), whereas 6 out of 37 old mice succumbed (Fisher exact test, *P* = 0.019). The mortality of old mice mostly occurred in the anti-IL-10R/LPS group (5/10), one individual (1/9) died in the IgG/PBS group, whereas none of the mice died in the anti-IL-10R/PBS (0/9) and the IgG/LPS (0/9) groups (Log-Rank, χ^2^_3_ = 15.01, *P* = 0.0018) (Figure 1).

### Body Mass

**Figure 2:**
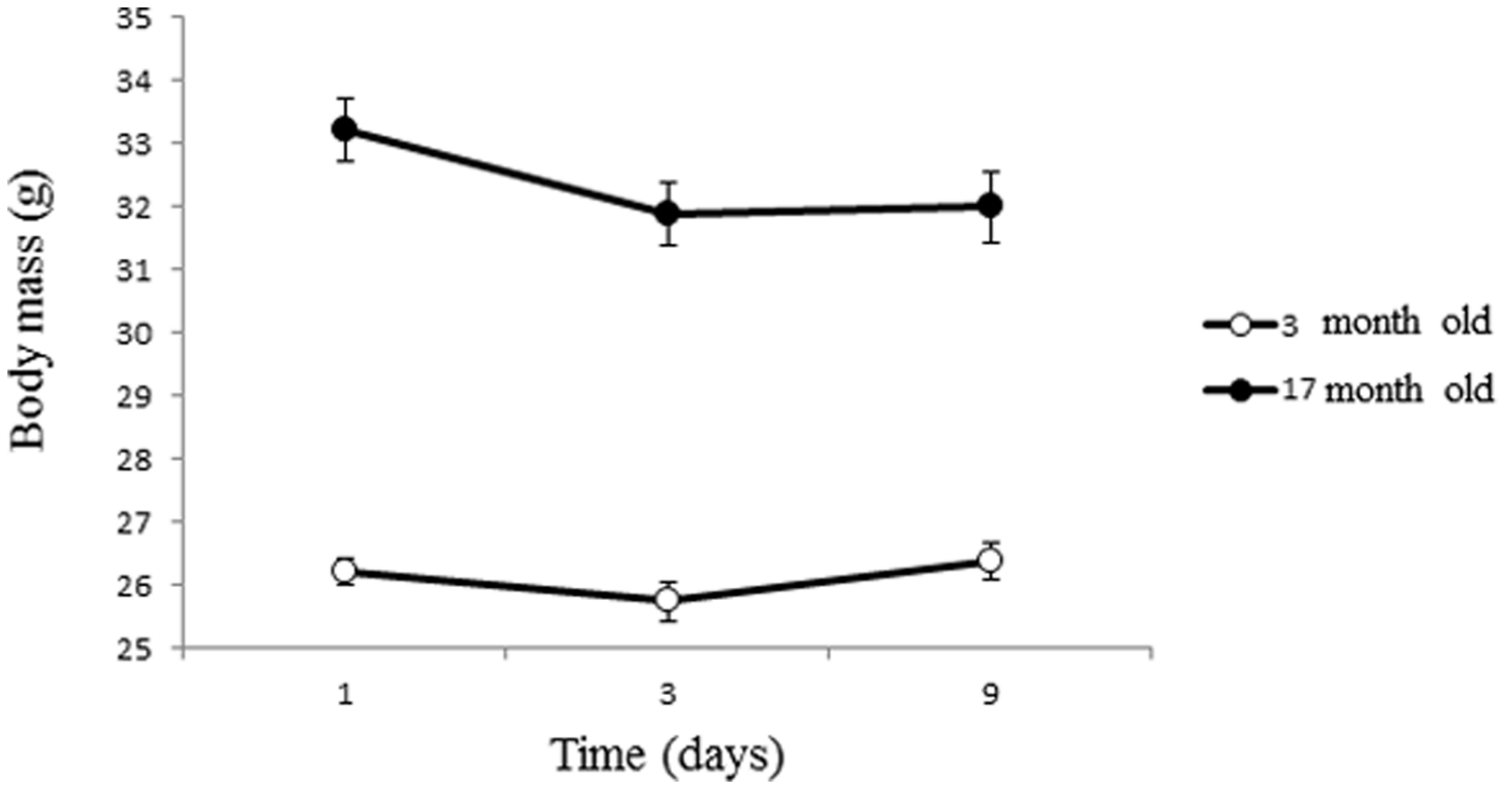
Change in body mass (g) during the course of the experiment for young (3 month old) and old (17 month old) male mice. We report means with standard errors.

Old mice had initially a much larger body mass than young individuals. However, they tended to lose body mass during the course of the experiment, whereas young mice maintained a constant body mass which resulted in a statistically significant age * time interaction (Table 1, Figure 2).

**Table 1:** Mixed linear model exploring the effect of the treatments and age on the changes in body mass over the nine days of the experiment.

*Sources of variation*	*df*	*F*	*P*
**anti-IL-10R/IgG**	1,64	3.15	0.0806
**LPS/PBS**	1,64	3.29	0.0745
**Age**	1,64	140.02	<0.0001
**Time**	2,64	17.41	<0.0001
anti-IL-10R/IgG* LPS/PBS	1,64	0.00	0.9562
anti-IL-10R/IgG *age	1,64	0.02	0.8790
LPS/PBS *age	1,64	0.52	0.4742
anti-IL-10R/IgG *time	2,64	10.34	0.0001
LPS/PBS *time	2,64	18.28	<0.0001
age*time	2,64	10.11	0.0002
anti-IL-10R/IgG * LPS/PBS *age	1,64	0.71	0.4025
anti-IL-10R/IgG * LPS/PBS *time	2,64	11.20	<0.0001
anti-IL-10R/IgG *age*time	2,64	1.58	0.2149
LPS/PBS *age*time	2,64	1.55	0.2206
anti-IL-10R/IgG * LPS/PBS *age*time	2,64	0.68	0.5088

Age: 3 vs 17 months. Individual identity was declared as a random factor.

**Figure 3:**
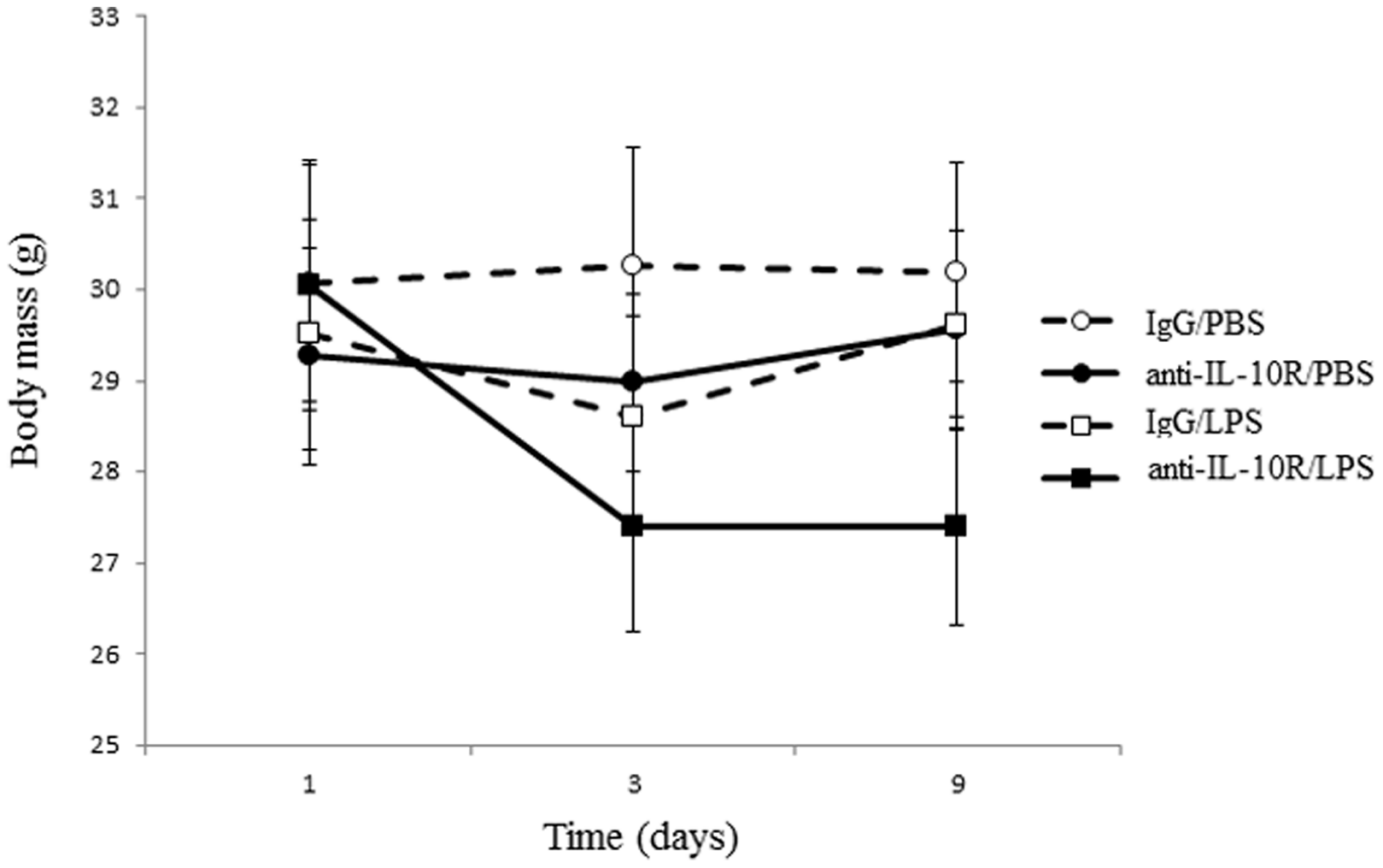
Change in body mass (g) during the course of the experiment for male mice according to the four experimental treatments. We report means with standard errors.

The two treatments affected the changes in body mass, independently of individual age. As expected, mice in the anti-IL-10R/LPS group showed the largest decline in body mass as shown by the three-way interaction between time and the two treatments (Table 1, Figure 3). None of the interactions between age and the treatments was statistically significant (Table 1).

Four months after the end of the experiment, we measured the body mass of mice that had not been sacrificed, to look at any possible long lasting effect of the treatments. This only involved young individuals that were therefore almost 8 month-old when they were measured again. Neither the anti-IL-10R nor the LPS treatments affected body mass after four months (anti-IL-10R, *F_1,16_* = 0.02, *P* = 0.8835; LPS, *F_1,16_* = 0.23, *P* = 0.6378; anti-IL-10R * LPS, *F_1,16_* = 0.03, *P* = 0.8681), suggesting that the effects were transitory.

### Testis Mass

Testis mass was analyzed in two steps. First, we analyzed right and left testis in separate models. Then, we included both variables in the same MANOVA model.

**Figure 4:**
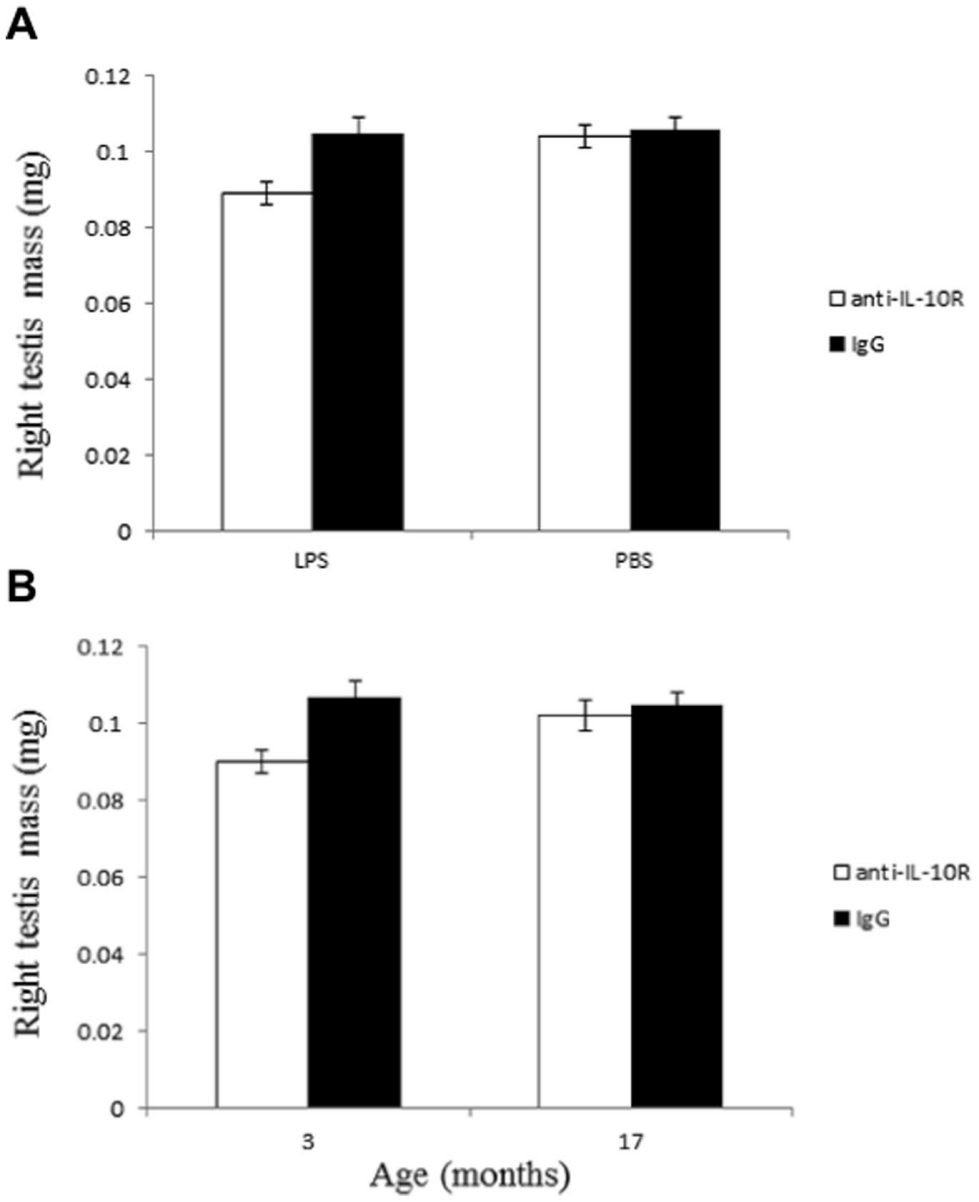
Effect of treatments and age on the right testis mass (mg). (A) Right testis mass according to the four experimental groups. (B) Right testis mass of young (3 month old) and old (17 month old) mice injected with the anti-IL-10R antibodies or the IgG control. We report means with standard errors.

The analysis of the right testis showed that testicular mass was affected by the interaction between the two treatments (*F_1,33_* = 4.46, *P* = 0.0424), and the interaction between age and the anti-IL-10R treatment (*F_1,33_* = 5.16, *P* = 0.0298). Mice with an impaired anti-inflammatory response injected with LPS had significantly smaller right testes compared to the other three groups (Figure 4a). Young mice had smaller right testes than old mice only when injected with the anti-IL10R antibodies (Figure 4b).

The model on the left testis confirmed that testicular mass was affected by the interaction between the two treatments (*F_1,33_* = 7.00, *P* = 0.0124), whereas age did not explain variation in left testis mass (main factor and interactions with the two treatments, all *P’s* >0.1).

The MANOVA model showed that the interaction between the two treatments (Wilks’ λ = 0.813, *F_2,31_* = 3.55, *P* = 0.0408) and the interaction between age * anti-IL-10R treatment (Wilks’ λ = 0.709, *F_2,31_* = 6.36, *P* = 0.0048) significantly affected testis mass.

As for body mass, these effects were transitory. Four months after the end of the experiment testis mass was unrelated to the treatments (right testis: anti-IL-10R, *F_1,16_* = 0.16, *P* = 0.6973; LPS, *F_1,16_* = 0.22, *P* = 0.6491; anti-IL-10R * LPS, *F_1,16_* = 1.55, *P* = 0.2309; left testis: anti-IL-10R, *F_1,16_* = 0.00, *P* = 0.9924; LPS, *F_1,16_* = 0.21, *P* = 0.6561; anti-IL-10R * LPS, *F_1,16_* = 0.67, *P* = 0.4265).

### Sperm Morphology

**Figure 5:**
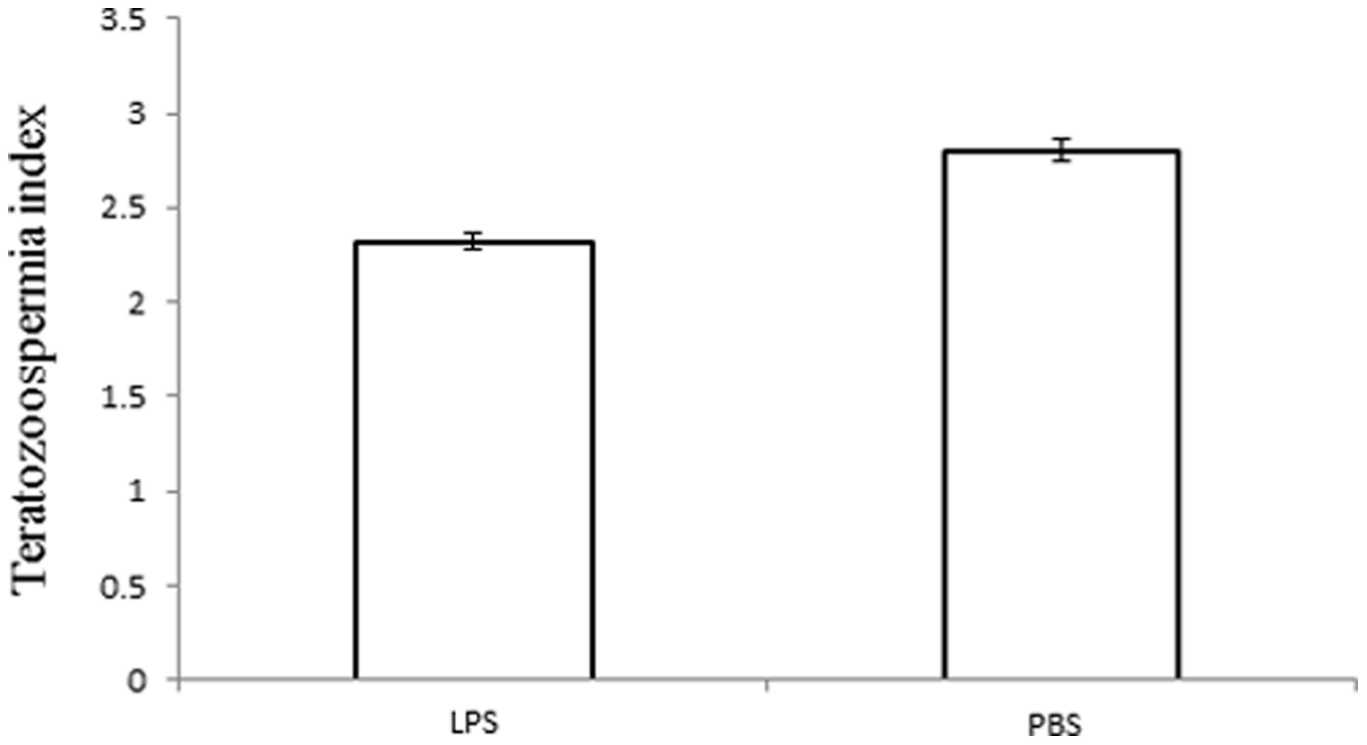
Teratozoospermia index for mice injected with LPS or PBS. We report means with standard errors.

Aberrant sperm morphology was assessed using MANOVAs. Sperm collected in the epididymis or in the testes were similarly affected by the experimental treatments, since only the LPS injection resulted in an increased proportion of aberrant morphology (Table 2 and 3, Figure 5).

**Table 2:** MANOVA exploring the effect of treatments and age on the morphology of sperm collected in the epididymis and the testes.

Epididymis					
*Sources of variation*	*Wilks’ λ*	*Df*	*F*	*P*	
**anti-IL-10R**	0.90	3,29	1.06	0.3833	
**LPS/PBS**	0.43	3,29	12.75	<0.0001	
**Age**	0.90	3,29	1.03	0.3957	
anti-IL-10R * LPS/PBS	0.90	3,29	1.12	0.3560	
anti-IL-10R *age	0.87	3,29	1.51	0.2338	
LPS/PBS *age	0.97	3,29	0.31	0.8172	
anti-IL-10R * LPS/PBS *age	0.82	3,29	2.12	0.1189	
**Testes**					
***Sources of variation***	***Wilks’ λ***	***Df***	***F***	***P***	
**anti-IL-10R**	0.99	3,30	0.12	0.9496	
**LPS/PBS**	0.63	3,30	5.83	0.0029	
**Age**	0.88	3,30	1.40	0.2610	
anti-IL-10R * LPS/PBS	0.92	3,30	0.87	0.4667	
anti-IL-10R *age	0.84	3,30	1.93	0.1456	
LPS/PBS *age	0.97	3,30	0.30	0.8275	
anti-IL-10R * LPS/PBS *age	0.96	3,30	0.46	0.7097	

Age: 3 vs 17 months. The dependent variables were the teratozoospermia index and the proportion of sperm with single and multiple morphological abnormalities.

**Table 3:** Morphology sperm collected in the testes of young and old mice according to the different experimental groups.

		Abnormal	Hairpin(%)	Sperm tail morphology (%)				Sperm with cytoplasmatic droplet (%)		Abnormal	TZI
Young		Spermhead (%)		Straight	Proximalbent	Distalbent	Coiled	Light	Heavy	Sperm (%)	
	Saline	17.2±3.9	1.2±0.5	60.0±4.7	32.0±3.7	4.4±1.2	4.4±1.2	11.4±4.1	10.2±2.4	51.8±3.1	1.61±0.13
	Saline	24.2±1.6	0.8±0.6	57.8±6.1	33.8±5.6	5.6±0.5	3.2±0.8	12.0±3.7	9.4±0.7	55.8±4.5	1.64±0.10
	LPS	22.4±3.4	1.0±1.0	49.2±6.6	40.6±4.6	2.6±0.9	7.6±2.1	7.8±2.5	15.8±3.2	62.0±5.2	1.63±0.11
	LPS	39.2±7.2	2.6±4.9	49.6±4.9	37.6±3.5	7.2±1.7	5.8±1.6	6.6±2.1	12.4±2.9	70.8±4.9	1.63±0.07
**Old**											
	Saline	26.0±0.8	2.2±1.1	55.8±6.1	34.4±4.3	4.0±0.6	6.4±2.5	11.6±3.4	8.6±1.5	60.6±3.9	1.61±0.04
	Saline	17.8±2.0	4.6±2.7	56.6±6.3	32.8±6.7	4.4±1.6	6.2±1.2	12.0±1.4	7.2±2.9	59.2±4.1	1.47±0.06
	LPS	34.6±1.8	2.6±0.7	50.6±2.8	38.2±3.8	5.0±1.1	7.2±1.8	8.8±3.1	7.8±1.2	74.6±3.3	1.53±0.05
	LPS	31.4±5.4	4.4±2.1	53.4±5.2	26.0±4.4	14.8±2.8	8.0±1.7	9.4±1.9	8.8±3.4	68.2±4.5	1.59±0.05

We report means ± SE, n = 5 mice per group. Young = 3 month old; Old = 17 month old. TZI: teratozoospermia index.

## Discussion

It is now well established that an overreacting or misdirected immune response can produce substantial damage to the host [16], [18]. This leads to the rather straightforward prediction that mechanisms that regulate the immune response or prevent the mis-targeting of host structures should play a prominent role to maintain organismal homeostasis during the infection. Nevertheless, up-to-date, few studies have explicitly addressed the fitness consequences of a disrupted immune regulation [21], [22], especially in terms of reproductive fitness.

Here, we showed that a phenotypic manipulation of the anti-inflammatory cytokine IL-10, produced a marked reduction of the male reproductive function in mice that were exposed to a mild inflammatory challenge. Males whose IL-10 was experimentally impaired by the injection of antibodies that block the IL-10 receptors had smaller testis when concomitantly exposed to a LPS stimulus. The disruption of the anti-inflammatory response also produced different results according to individual age, since young males injected with the anti-IL-10R antibodies had the smallest testis size. The treatment-induced reduction in testis mass was very fast as it occurred over a time of 9 days, and it was transitory since testis mass measured four months after the end of the experiment was unrelated to the experimental treatments.

Testis size can be considered a good proxy of investment into sperm production since testicular weight has been shown to explain up to 80% of variation in daily sperm production [39], [40]. Of course, testis size does not perfectly correlates with sperm production because it integrates both spermatogenic and non-spermatogenic tissues (i.e., tunica albuginea and Leydig cells). Changes in spermatogenic and non-spermatogenic tissues may occur independently from each other adding noise to the relationship between sperm production and testis size. Spermatogenic efficiency refers to the production of sperm per unit of testicular tissue and it has been shown that artificial selection for bigger testis size also induced higher spermatogenic efficiency in pigs [41]. This relationship seems rather general since marine flatworms (*Macrostomum lignano*) experimentally induced to have bigger testes have a higher rate of sperm production [40]. Further evidence shows that variation in testis size does not only affect sperm production but also siring success, since male Soay sheeps with larger testes have a better reproductive success [42], possibly because, in addition to the benefit in terms of sperm production, individuals with larger testes suffer less from sperm depletion [43].

Given the relatively tight association between testes size and the sperm production rate, testes mass has been repeatedly used in sperm competition studies. Interspecific comparative work has shown that species with a mating system that gives raise to sperm competition have larger testes that species where the risk of sperm competition in low [44], [45]. Even at the within species level, risk of sperm competition and testes size do correlate. Firman and Simmons [46] studied seven insular populations of the house mouse (*Mus domesticus*) that vary in the degree of multiple paternity (a proxy of population specific risk of sperm competition). They found that testis size positively covaried with multiple paternity across islands. Testis size is therefore an important component of male fitness especially in species where males are under the threat of paternity loss due to sperm competition like in house mice. Our finding of a reduced testis mass in individuals injected with LPS and with an impaired immune response could therefore translate into a substantial fitness cost in terms of siring success.

Interestingly, the LPS alone did not induce any loss in testicular mass but considerably increased the number of sperm with abnormal morphology. Sperm morphology is also an important determinant of siring success; therefore, the LPS injection, although involving a very mild dose, might compromise reproductive fitness. LPS is a potent activator of the inflammatory response. LPS binds to Toll-like receptors (TLRs) and induces the activation of inflammatory cells which release reactive oxygen and nitrogen species. Previous work has clearly established that infection can compromise testicular function, inducing a temporary reduction in male fertility. For instance, a systemic inflammation triggered by LPS injection in rats affected the seminiferous epithelium with pronounced effects on stages IX-XIII of spermatocytes [47]. Experimental evidence shows that *in vivo* injection of different doses of LPS impair steroidogenesis (testosterone production) but also induce spermatogenic damage [33]. Pro-inflammatory cytokines and nitric oxide might be involved in this process [32]. For instance, Zhang et al. [48] have recently shown that the expression of TLRs and pro-inflammatory cytokines (IL-1β, IL-6) was up-regulated in the testes of roosters injected with LPS.

It also generally thought that testis are “immune privileged” because i) they tolerate allografts, and ii) haploid gametic cells should not be recognized as “self” by the diploid cells of the immune system [49], [50]. To maintain testicular functioning and homeostasis, several immune regulatory mechanisms operate in the testis, down-regulating both adaptive, antigen-specific responses, and innate responses [50]. For instance, it has recently been shown that resident testicular macrophages have a constitutively high production of the anti-inflammatory cytokine IL-10 in vitro [51]. Our in vivo experimental manipulation of IL-10 by the mean of the anti-IL-10 receptor antibodies corroborates the view that disrupting this regulatory pathway can produce substantial testicular damage. Surprisingly, however the sperm morphology was only affected by the LPS injection, and even in the group of mice with a normal immune regulation, the LPS exposure produced a deterioration of sperm morphology. The fitness consequences of altered sperm morphology are likely to be a reduced fertilization power of these ejaculates and perhaps heritable genetic defects for the progeny.

The disruption of the anti-inflammatory machinery (at least of the IL-10) produced a cost in terms of male reproductive function that adds up to the survival and body mass cost. Survival and body mass were much reduced in individuals simultaneously injected with the LPS and the anti-IL-10R compared to the other groups. However, the survival cost was only appreciable in old individuals, whereas the reduction in body and testis mass occurred in both age classes. Age-specific survival costs of disrupted immune regulation were already reported in a previous experiment where we used mice of similar age classes [22]. The absence of age-specific response to the disruption of the inflammatory response in terms of male function, at least in the predicted direction (old individuals suffering more than young ones) was somehow surprising. Aging is often associated with both a decline in reproductive function and a pro-inflammatory status [52]–[56]. We therefore predicted that enhancing the pro-inflammatory status might exacerbate the decline in testicular function relatively more in senescing individuals than in young adults. One possible explanation might be that our old individuals were not senescing yet. However, there was a very clear-cut difference between young and old individuals in terms of survival profile. Therefore, the most plausible explanation is that the treatment selectively sorted out old individuals that better tolerated the concomitant activation of the inflammatory response and the disrupted immune regulation, since testis mass and sperm morphology was only assessed in surviving mice. This selective effect could also explain why testis mass of young males injected with the anti-IL-10R antibodies was lower than testis mass of older individuals.

To conclude, we have provided here evidence in support to the idea that immune regulation confers protection against the risk of inflammation-induced infertility during infection. Since we used a very mild inflammatory insult, we believe that, even though our experiment involved a laboratory strain of mice, our results might apply to a more natural context where animals have to face persistent infection threats during their life time.
